# The impact of restoration protocols on the fracture resistance of root canal treated anterior teeth: an in vitro study

**DOI:** 10.1007/s44445-026-00149-9

**Published:** 2026-04-02

**Authors:** Samar Yasir Ahmed Mohamed, Mohamed Adel Eldemellawy, Fatma Adel Mohamed Ahmed

**Affiliations:** Department of Fixed Prosthodontics, Faculty of Dentistry, Ain Shams University, Organization of African Unity Street, El-Qobba Bridge, El Weili, Cairo, Egypt

**Keywords:** Fracture resistance, Fiber post, Intraradicular restoration, Composite core material, Endodontically treated anterior teeth

## Abstract

This study aimed to evaluate the fracture resistance of endodontically treated maxillary central incisors restored with various post-and-core configurations and computer-aided design/computer-aided manufacturing (CAD/CAM) resin composite crowns. Additionally, it assessed whether restoration design influences mechanical performance. Twenty-one maxillary central incisors underwent endodontic treatment and were allocated into three groups (*n* = 7). The FRCP group received a fiber-reinforced composite (FRC) post with a composite core; the NRCC group received a nanoparticle zirconia-reinforced composite core alone; and the FRCC group received an FRC core alone. Following fabrication of CAD/CAM resin-composite crowns, the specimens underwent 10,000 thermal cycles. Specimens were loaded to failure in a universal testing machine, and fracture patterns were examined under a stereomicroscope. Restoration type significantly influenced fracture resistance (*p* = 0.014). Mean fracture loads were 612.36 ± 87.80 Newtons (N) for FRCP, 516.98 ± 80.34 N for NRCC, and 465.65 ± 84.95 N for FRCC. The fiber post–composite core configuration exhibited higher fracture resistance compared to composite core materials alone. However, all groups withstand loads exceeding typical anterior biting forces, suggesting that reinforced composite cores may serve as viable alternatives for restoring endodontically treated maxillary central incisors.

## Introduction

The long-term prognosis of endodontically treated teeth depends primarily on the amount of remaining tooth structure (Comba et al. 2021; Iemsaengchairat and Aksornmuang 2021; Li et al. 2024; Ng et al. 2006). When coronal structure is largely intact and loading conditions are favorable, such as in anterior teeth positioned distal to the occlusal fulcrum, direct restoration of the access cavity may suffice (Rosenstiel et al. 2015). However, significant loss of coronal structure compromises the tooth's ability to resist functional stresses, particularly lateral and tipping forces. In such cases, restorative strategies often necessitate an intraradicular post, core, and crown to re-establish form and function (Casanova and Özcan 2021). The primary function of the intraradicular post is to retain the core and final restoration, relying on root dentin for anchorage (Casanova and Özcan 2021; Iemsaengchairat and Aksornmuang 2021; Lassila et al. 2020).

Ideally, the elastic properties of restorative materials should match those of dentin. Metal posts exhibit a considerably higher modulus of elasticity than dentin, predisposing teeth to unfavorable stress accumulation and increasing the risk of catastrophic root fracture (Casanova and Özcan 2021; Iemsaengchairat and Aksornmuang 2021; Pham and Huynh 2019). Fiber-reinforced composite (FRC) posts offer an alternative; they address esthetic concerns in anterior teeth and possess an elasticity more comparable to dentin, facilitating advantageous stress distribution (Casanova and Özcan 2021; Garoushi et al. 2009; Pham and Huynh 2019; Santos et al. 2022).

Despite these benefits, post-debonding and loss of retention remain prevalent clinical failures, and root fractures are not entirely eliminated by FRC posts (Casanova and Özcan 2021; Iemsaengchairat and Aksornmuang 2021). Furthermore, treatment effectiveness is compromised when residual coronal structure is severely damaged, as the remaining tooth may be too fragile to withstand masticatory forces (Iemsaengchairat and Aksornmuang 2021; Ng et al. 2006). Fracture resistance in endodontically treated teeth is significantly influenced by residual root dentin thickness (Iemsaengchairat and Aksornmuang 2021; Saad et al. 2023). Excessive preparation of the cervical post space can leave the root surrounded by a thin dentinal wall (Faria-e-Silva et al. 2009).

While lengthening the post improves retention, bonding effectiveness in the apical region remains unpredictable (Liang et al. 2007; Schiavetti et al. 2010) due to the technical difficulty of performing multiple cementation steps, such as etching, drying, and adhesive application, precisely within the intraradicular space (Aguiar et al. 2011; Behr et al. 2008; Chung et al. 2009; Ho et al. 2011). Advances in adhesive dentistry have promoted less invasive approaches prioritizing tooth structure conservation. The use of self-adhesive resin cement simplifies cementation, improves fracture resistance, and strengthens the bond between short FRC posts and radicular dentin (Casanova and Özcan 2021; Pereira et al. 2013, 2021). Evidence suggests that shorter FRC posts may yield restorable fracture patterns and increased fracture resistance compared to longer posts, attributed to increased residual dentin thickness and enhanced adhesive integrity (Jakubonytė et al. 2018; Zicari et al. 2012).

Resin composite is frequently used for core build-ups due to its hardness and fracture toughness, which are comparable to natural tooth structure, allowing for light-curing treatment (Combe et al. 1999; Grandini et al. 2005). Recently, specific core build-up composites with increased filler content have been developed to enhance strength and handling. Variations among these materials include viscosity, curing mode, filler percentage, and composition. Current techniques also include composites reinforced with various fibers; the efficacy of this reinforcement depends on stress transfer from the matrix to the fibers, governed by parameters such as resin type, fiber length, quantity, orientation, adhesion, and infiltration. (Garoushi et al.^2009^, ^2017^; Lassila et al. 2020).

Reinforced resin composite materials are being explored as substitutes for FRC or metallic posts to minimize root fractures, preserve tooth structure, and eliminate the need for luting cement (Casanova and Özcan 2021; Garoushi et al. 2009; Iemsaengchairat and Aksornmuang 2021; Lassila et al. 2020; Rayyan 2014). However, literature on this topic remains scarce. Therefore, the current investigation aimed to compare fracture resistance and failure mechanisms in anterior teeth restored with different systems, including a standard fiber post-and-core system and a reinforced resin composite core material alone. The null hypothesis posited that there would be no significant differences in fracture resistance or failure mode among the three experimental groups.

## Materials and methods

This study was approved by the Research Ethics Committee of the Faculty of Dentistry at Ain Shams University, Cairo, Egypt (FDASU-REC; Approval No. (FDASU-Res EM042205). Materials utilized in this investigation are listed in "Table 1". A power analysis was conducted to ensure adequate statistical power to test the null hypothesis that there is no difference in fracture resistance across the tested groups. Based on prior research findings (Casanova and Özcan 2021), the analysis utilized an alpha (α) of 5%, a beta (β) of 20% (power = 80%), and an effect size (f) of 0.905. The estimated sample size was 18 (6 per group); however, 21 samples were ultimately used, with 7 per group. Sample size calculation was performed using G*Power version 3.1.9.2.

**Table 1:** The materials employed throughout the investigation are presented with their main composition and manufacturer

Material name	composition	Manufacturer
Build-it FR core buildup material	Bis-GMA, UDMA, HDDMA, 67.3 wt%, barium Boro-aluminosilicate glass fillers and chopped Glass Fiber	PENTRON USA
Luxacore Z-dual Core buildup material	Bis-GMA, Barium glass, Pyrogenic silicic acid, Nano fillers and Zirconium oxide	Hamburg-Germany
BisCem self-adhesive resin cement	Base: Bisphenol-A glycidyl Dimethacrylate, uncured Dimethacrylate monomer and Glass filler Catalyst: Phosphate Acidic monomer and Glass fillers	Bisco-USA
Palfique universal bond	Bond A: Adhesive monomer; new 3D-SR monomer (phosphoric acid monomer), (MTU-6 and γ-MPTES), (HEMA, Bis-GMA, and TEGDMA) Bond B: Acetone, isopropyl alcohol and water Borate and peroxide	Tokuyama-Japan
SILAN-IT Silane	-Ethanol −96% -Silane −4%	Itena Paris—France
FineEtch phosphoric acid	37% phosphoric acid Gel	Spident, Korea

### Teeth selection and preparation

**Fig. 1:**
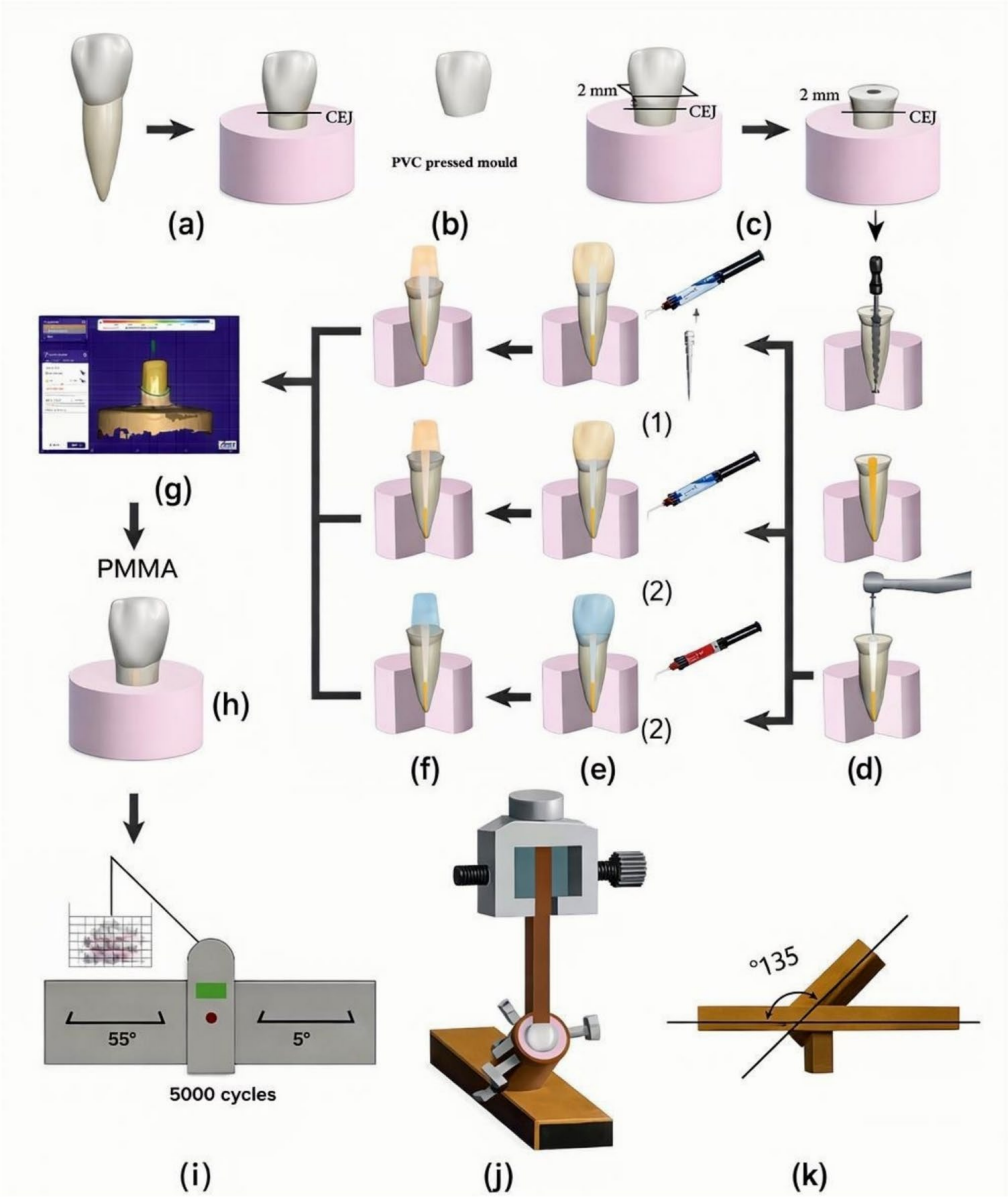
Graphic diagram for the methodology procedure. a: Sound upper central mounted in acrylic resin blocks. b: Transparent PVC (Poly-vinyl chloride) mould. c: Sample decoronation 2 mm incisal to the CEJ. d: Endodontic procedure and post space preparation. e: Post and core fabrication (1) FRCP group (2) NRCC group (3) FRCC group. f: Crown preparation. g: Restoration design. h: PMMA crown fabrication. i: Thermocycling. j: fracture test. k: Special attachment with 135º inclination

Twenty-one maxillary central incisors were selected from a collection of extracted teeth obtained for medical reasons or orthodontic clearance. The teeth exhibited straight roots, measured 14–16 mm in length, and were free of cavities, fractures, defects, or previous restorations. Soft tissue and calculus were manually removed using an ultrasonic scaler (Suprasson P5 Booster, Satelec, Merignac, France), and the teeth were immersed in 70% ethanol for 24 h before being preserved in distilled water at room temperature for the duration of the experiment. For standardized handling, each tooth root was embedded vertically in epoxy resin blocks (CMB, Egypt), with the long axis aligned vertically "Fig. 1a". One sample was used to make a mold for the following construction of a composite core by taking impression using addition silicone (Zhermack Elite HD +, Italy), which was then poured into type IV extra hard stone (TST, China) to generate a die. A soft transparent polyvinyl chloride sheet (Smile Find, Henan, China) was then vacuum-pressed over the stone die to form the mold "Fig. 1b".

A low-speed diamond disc (Giflex, Bredent, Senden, Germany) was used to decoronate the teeth approximately 2 mm coronal to the cemento-enamel junction "Fig. 1c". Endodontic preparation was prepared with rotary instruments (M-Pro, AlexDent, China) up to a #35 file with a 0.04 taper, terminating 0.5 mm shorter than the root apex "Fig. 1d". During instrumentation, canals were irrigated with 5% sodium hypochlorite solution (JK Dental, Egypt). Canals were dried using paper points (Meta Biomed, Korea); obturation was performed using a size 35 single master cone and bioceramic sealer (CeraSeal, Meta Biomed, Korea) "Fig. 1d".

Post spaces were prepared using a white drill (DentoClic, Itena, Paris, France) to a depth of 8 mm within the canal, leaving approximately 5 mm of intact apical gutta-percha "Fig. 1d". Following post-space preparation, all specimens were rinsed with 5% sodium hypochlorite and saline solution, then dried with an air syringe and absorbent paper points. Specimens were then treated for 15 s with 35% phosphoric acid (FineEtch 37, Spident, Korea), rinsed with water for 15 s, and dried with an air syringe; absorbent paper points were used to achieve total dryness. Both the intraradicular dentin and coronal portion of the tooth were treated with a universal adhesive (Palfique Universal Bond, Tokuyama, Japan) using a micro-applicator (UNIPACK, China). One drop of adhesive components A and B was dispensed, thoroughly mixed with a disposable applicator, and applied within one minute. After application, a gentle air stream was used to achieve a uniform film thickness for 30 s. Paper points were used to remove excess adhesive. Subsequently, the samples were randomly divided into three groups.

### Post and core fabrication

**FRCP Group:** In this group, roots were treated with white glass fiber posts (Ø 1.2 mm, DentoClic, Itena, Paris, France), trimmed 4 mm coronal to the CEJ with a high-speed handpiece (Coxo, China) and a medium-grit bur (Diamond Bur 850 C, China). The post surfaces were cleaned using an alcohol-soaked cotton pellet before being coated with silane (SILAN-IT, Itena, Paris, France). After a gentle air stream to remove solvent, the post was cemented into the canal with a dual-cured self-adhesive cement (Biscem, Bisco, USA), applied according to the manufacturer's recommendations. Mild finger pressure was used when placing the post in the canal. A core build-up material (LuxaCore Z, DMG, Hamburg, Germany) was then applied within a prefabricated PVC mold and placed over the decoronated teeth. Initial light-curing (Woodpecker LED Curing Light, China) for 5 s facilitated easy removal of excess core material with a dental probe, followed by final curing for an additional 40 s from all directions; labial, palatal, mesial/distal "Fig. 1e1".

**NRCC Group:** A nanoparticle zirconia-reinforced composite core material (LuxaCore Z-Dual, DMG, Hamburg, Germany) was injected into the canal using a small intraoral tip. A portion of the material was also injected into the PVC mold, which was then placed over the center of the decoronated tooth. Photopolymerization was performed identically to the FRCP group "Fig. 1e2".

**FRCC Group:** A FRC core material (Build IT, Pentron, USA) was utilized as both post and core material and processed in the same manner as the NRCC group "Fig. 1e3".

### Crown preparation and construction

**Fig. 2:**
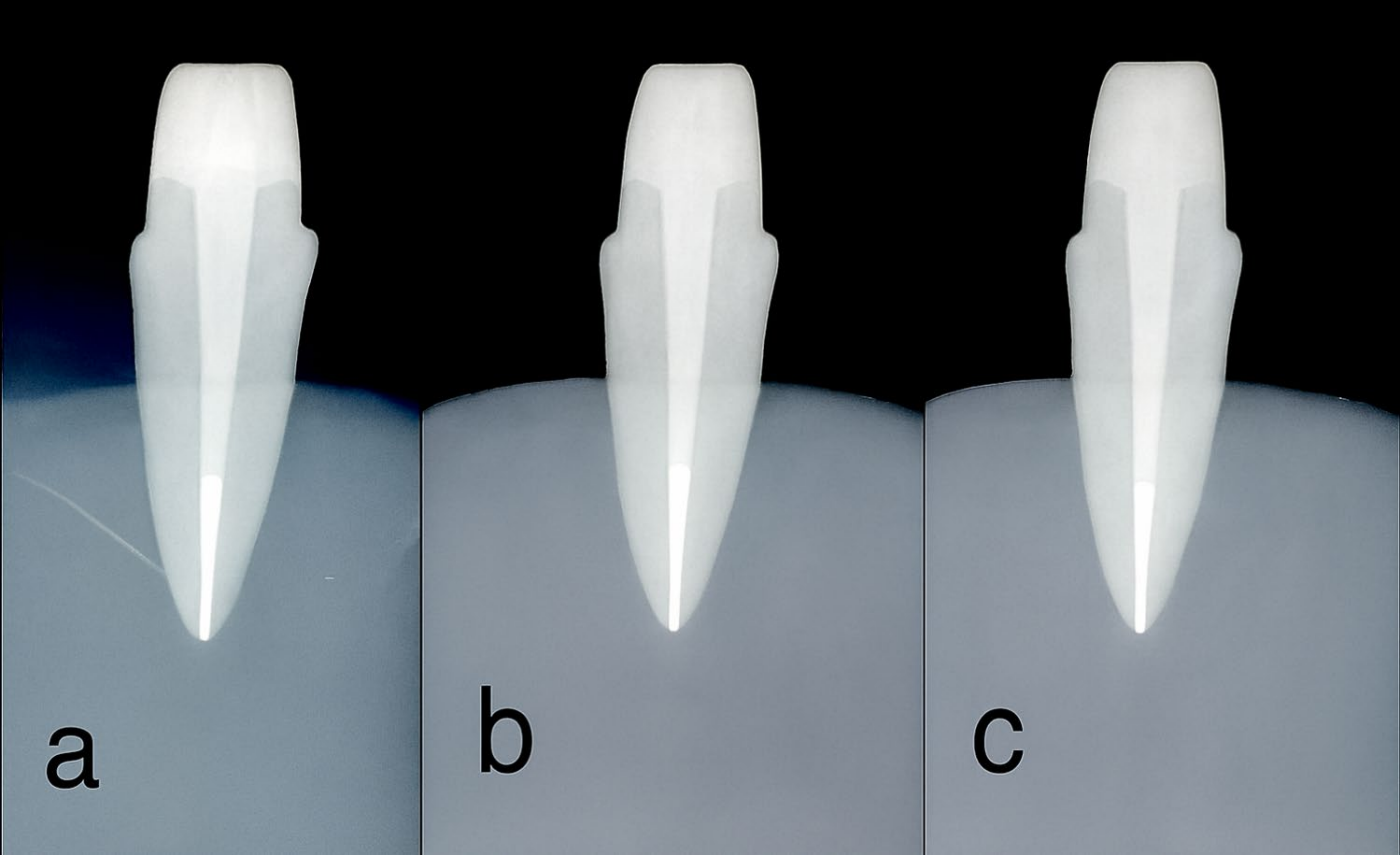
Radiographic image for the three groups after preparation. **a**: FRCP group. **b**: NRCC group. **c**: FRCC group

To achieve consistent preparation dimensions, a surveyor (NOUVAF, AF30, Switzerland) was used to prepare a 0.5 mm rounded chamfer finish line at the coronal limit of a 2 mm ferrule on each tooth "Fig. 1f". All specimens were radiographed to verify completion of the procedure "Fig. 2". The preparations were subsequently scanned using a desktop scanner (DOF Inc., Seoul, South Korea); the scanned data were converted into Standard Triangulation Language (STL) files and uploaded to CAD software (Exocad DentalCAD, version 3, Exocad GmbH, Darmstadt, Germany), "Fig. 1g". Computer-aided design/computer-aided manufacturing (CAD/CAM) resin composite crowns were fabricated using a milling machine (Imes-Icore GmbH, Germany) and polymethyl methacrylate (PMMA) discs (Aidite PMMA, China). After the crowns were silanized, they were cemented to the prepared samples with a dual-cured self-adhesive resin cement "Fig. 1h".

### Fracture testing and thermal cycling

The samples underwent 10,000 thermocycles between 5 °C and 55 °C using a thermocycler (SD Mechatronic, Germany) *"*Fig. 1i*".* Subsequently, the samples were mounted in a universal testing machine (Model LR5K; Lloyd Instruments Ltd, Fareham, UK) at an angle of 135° using a customized device *"*Fig. 1k*"*. A force was delivered at an angle of 45° relative to the tooth axis, using a load applicator placed on the palatal side, 3 mm apical to the crown's incisal edge. Loading continued until fracture occurred *"*Fig. 1j*".*

### Assessment of mode of failure

A digital microscope (Dino-Lite, Taiwan) with a built-in camera, mounted on a precision microscopic stand, was connected to a computer and set to a fixed magnification of 45 × to examine the fractured specimens and fragments. The fracture patterns were categorized based on the location of the fracture line as either irreparable or reparable. An irreparable fracture extends apical to the CEJ, while a reparable fracture terminates coronal to the CEJ (Comba et al. 2021). Additionally, failures were classified into the following categories (Iemsaengchairat and Aksornmuang 2021): Class 1 included fractures of the post and/or core, whereas Class 2 referred to root fractures occurring in conjunction with post and/or core fractures.

### Statistical analysis

Fisher's exact test was used to examine categorical data, which were reported as percentages and frequencies. The Shapiro–Wilk and Levene's tests were used to assess homogeneity of variance and normality of the numerical values, which were reported as means and standard deviations (SD). One-way analysis of variance (ANOVA) and Tukey's post hoc test were used to evaluate normally distributed data with homogeneous variances. The maximum likelihood estimation (MLE) method was used to estimate the Weibull distribution parameters. A significance level of p < 0.05 was considered statistically significant. All statistical analyses were conducted using R software, version 4.3.3 for Windows.

## Results

### Fracture resistance (N)

The results revealed statistically significant differences among the groups (p = 0.014)** "**Table 2**"**. The FRCP group exhibited the highest mean fracture resistance (612.36 ± 87.80 N), followed by the NRCC group (516.98 ± 80.34 N) and the FRCC group (465.65 ± 84.95 N). Moreover, the FRCP group had significantly higher fracture resistance than the FRCC group (p < 0.001). No statistically significant differences were observed between the FRCP and NRCC groups or between the NRCC and FRCC groups.

**Table 2:** Intergroup comparisons, mean and standard deviation (SD) values of fracture resistance (N)

Fracture resistance (N) (Mean ± SD)			*p*-value
FRCP	NRCC	FRCC	
612.36 ± 87.80^A^	516.98 ± 80.34^AB^	465.65 ± 84.95^B^	0.014*

### Weibull analysis

**Fig. 3:**
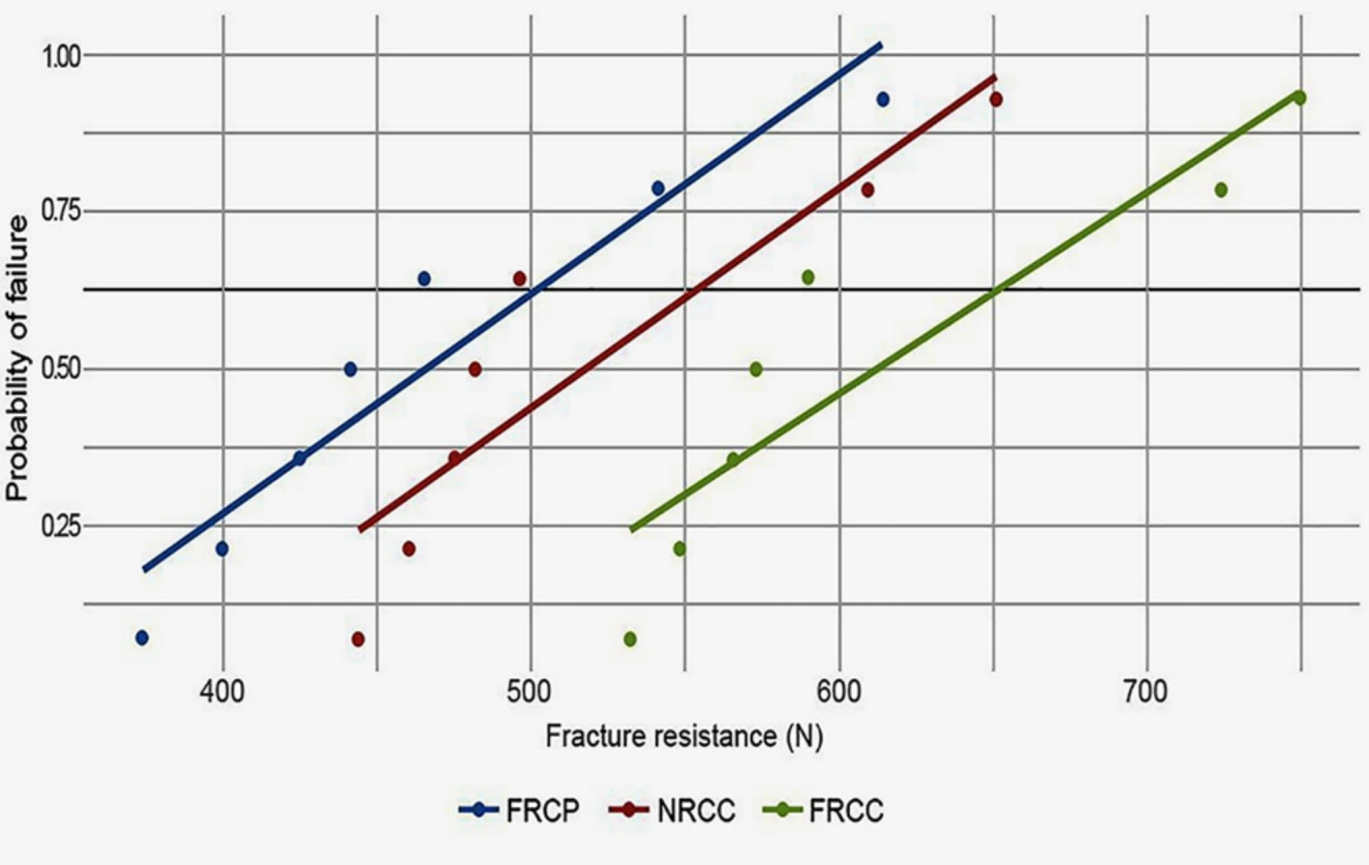
Probability plot

"Fig. 3" displays Weibull distribution parameters for each group. The FRCP group exhibited the highest Weibull modulus (7.60), indicating superior structural reliability, as well as the highest characteristic strength (649.82 N), suggesting a longer predicted lifespan. The NRCC group ranked second, with a modulus of (6.97) and a characteristic strength of 550.80 N. In contrast, the FRCC group had the lowest values for both modulus (6.02) and strength (500.09 N), indicating the least structural reliability and the shortest expected lifespan.

### Mode of failure

**Fig. 4:**
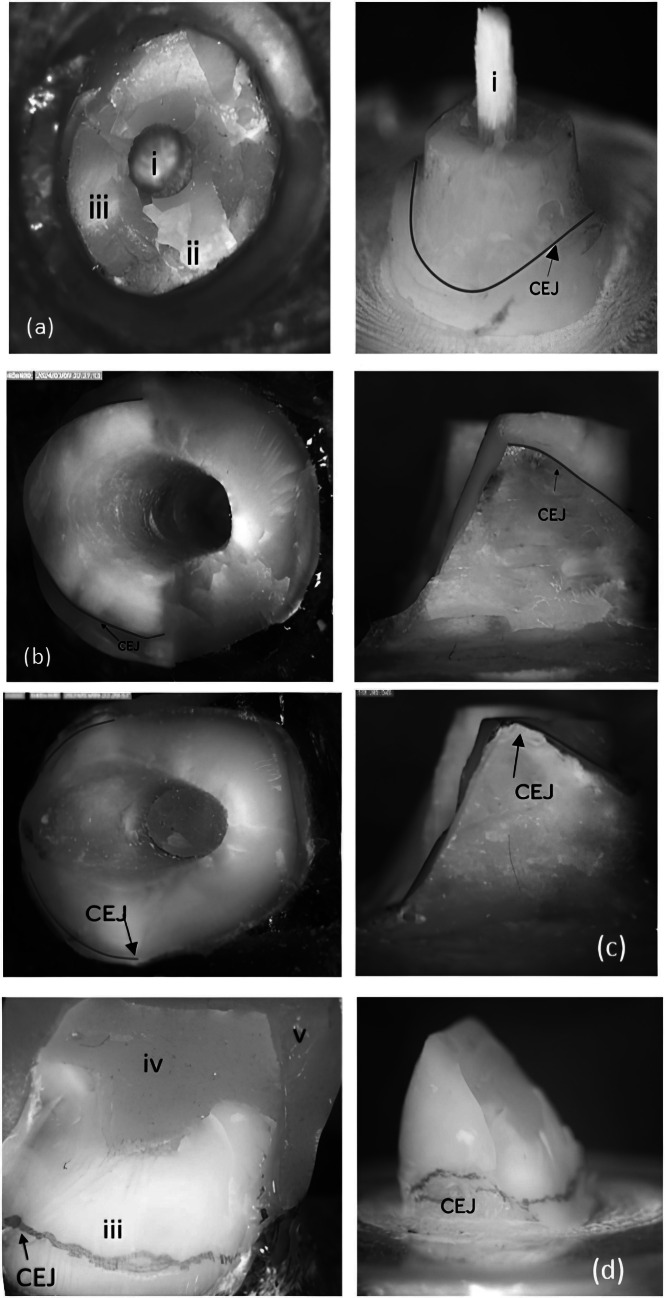
(**a**): FRCP group repairable fracture pattern (post and core fracture). (**b**): FRCP group irreparable fracture pattern (post, core and root fracture). (**c**): NRCC group irreparable fracture pattern (post, core and root fracture). (**d**): FRCC group irreparable fracture pattern (post, core and root fracture). i: fiber post, ii: cement remnant, iii: tooth structure, iv: core material, v: CAD/CAM resin composite crown, CEJ: cemento-enamel junction

The frequency and percentage of failure modes for each group are detailed in "Table 3", and representative images are provided in "Fig. 4". In the FRCP group, two specimens exhibited repairable fractures (post and/or core), while the remaining specimens displayed irreparable fractures (root fractures combined with post and/or core). There were no significant differences in failure mode distribution among the groups (p = 0.300).

**Table 3:** Intergroup comparisons, frequency, and percentage values for mode of failure

Mode of fracture	*n* (%)			*p*-value
	FRCP	NRCC	FRCC	
Repairable Post and/or core fracture	2 (28.57%)	0 (0.00%)	0 (0.00%)	0.300ns
Irreparable Root fracture combining with a post and/or core fracture	5 (71.43%)	7 (100.00%)	7 (100.00%)

## Discussion

The present study evaluated the influence of different restoration protocols on the fracture resistance of endodontically treated maxillary central incisors. Additionally, it investigated whether a reinforced resin-composite core material alone could replace a conventional fiber post. The results demonstrated that post-and-core build-up techniques significantly affected fracture resistance; therefore, the null hypothesis was rejected.

This investigation employed a static loading-to-fracture test to evaluate the effectiveness of various materials and restoration methods. Static loading simulates trauma scenarios in clinical practice, where impact forces can lead to complete restorative failure (Fadag et al. 2018; Naumann et al. 2006a, b). Anterior teeth are more susceptible to failure than posterior teeth due to the transverse nature of occlusal forces (Comba et al. 2021; Naumann et al. 2006a, b, 2008). Anterior teeth are more susceptible to failure than posterior teeth due to the transverse nature of occlusal forces (Gale and Darvell 1999; de V Habekost et al. 2007). In this study, 10,000 cycles were applied to approximate one year of clinical service (Munck et al. 2005).

The remaining tooth structure coronal to the CEJ plays a critical role in determining the failure threshold (Sorensen and Engelman 1990). Libman and Nicholls investigated the effect of different ferrule heights (0.5 to 2.0 mm) on the force-bearing capacity of teeth restored using cast posts and cores (Libman and Nicholls 1995). Their findings indicated that a minimum ferrule height of 1.5 mm is required for favorable treatment outcomes. Consequently, a 2-mm ferrule height was standardized in the current investigation. A CAD/CAM resin-based provisional crown was used (Casanova and Özcan 2021), allowing the primary focus to remain on evaluating the durability of the post-and-core material and failure mechanisms, independent of the crown material.

The core materials used in the current study were chosen for their handling properties, mechanical characteristics, and ease of manipulation. Compressive strength is crucial for core materials, as they often replace a significant portion of tooth structure. These materials must withstand intraoral compressive and tensile stresses during functional and parafunctional activities (Saygılı and Sahmali 2002). The materials employed in this study were Build-It FR and LuxaCore Z dual-cure composite cores. According to the manufacturer, Build-It FR is a fiber-reinforced composite containing 67.3% fillers by mass, with a compressive strength of 300 MPa. LuxaCore Z composite core material contains 70% filler and has a compressive strength of 380 MPa.

The current study found that most failures were irreparable, with only 2 specimens (28.6%) in the FRCP group experiencing reparable failure modes. The FRCP group exhibited the highest fracture resistance, likely due to the fiber post's ability to enhance load transfer between dentin and restorative materials (Comba et al. 2021; Saad et al. 2023), resulting in a more uniform stress distribution. These findings are consistent with prior investigations by Lippo lassila et al. and Casanova et al., which compared the fracture strength of posts and cores versus that of core materials alone (Lassila et al. 2020; Casanova and Özcan 2021). They concluded that, for endodontically treated anterior teeth, incorporating a fiber post improves static load-bearing capacity compared with core material alone, particularly in cases with significant coronal tissue loss. However, a study by Pham KV et al., reported no significant difference between restorations with posts and cores and those without (Pham and Huynh 2019). This discrepancy may be attributed to methodological differences. Specifically, the post-space depth in the post-less technique was shallow (4 mm), enhancing light-curing efficiency when Smart Dentin Replacement (SDR) was used. This likely led to better polymerization and stronger core bonding, potentially reducing the need for additional reinforcement. Furthermore, the aging protocols differed; Pham and Huynh applied only 500 thermocycles, whereas the current study employed 10,000. Extended thermal cycling is more likely to reveal weaknesses in bonding, stress distribution, and overall structural integrity.

Conversely, the findings differ from those of M. Rayyan, and Garoushi et al. who reported superior fracture resistance when core material was used alone without adding a fiber post. Variations in material and aging processes may explain these inconsistencies (Rayyan 2014; Garoushi et al. 2009).

In the current study, the NRCC group demonstrated greater fracture resistance than the FRCC group, potentially due to the higher filler content in the NRCC material, which increases its flexural modulus and, in turn, its fracture resistance. This aligns with Nakade et al., who reported that LuxaCore Z exhibited superior fracture toughness. This performance may be attributed to its unique composition and nanotechnology, which reduces particle agglomeration during manufacturing (Nakade et al. 2024).

Garoushi et al. assessed five commercial short-FRCs and found that the Build-It FR core material had the lowest mechanical performance, including fracture toughness and reinforcement capability (Garoushi et al. 2017). This supports the current investigation's outcome, which found that the FRCC group exhibited significantly lower fracture strength than the other groups. This weakness was likely explained by microstructural limitations, particularly the low quantity of reinforcing microfibers and fine filler particles. Consequently, its capacity to absorb and redistribute stresses was compromised.

Finally, comparing these results to the maximum biting force in the anterior region, which typically does not exceed 200 N (Chauhan et al. 2019; Rezaei Dastjerdi et al. 2015), suggests that all tested restoration techniques are suitable for repairing endodontically treated anterior teeth with significant coronal destruction.

## Limitations

A primary limitation of this study is the absence of mechanical fatigue loading, as only thermal cycling was performed to simulate aging. Future research should evaluate a wider range of core materials, post-and-core designs, and mechanical aging procedures to better understand the long-term efficacy of these restorative options under dynamic occlusal conditions.

## Conclusions

Within the limitations of the current investigation, teeth restored with fiber posts and composite core materials exhibited greater load-bearing capacity than those restored with composite core materials alone. Although the FRCP group exhibited a higher frequency of reparable fracture patterns, this difference was not statistically significant.

## Clinical relevance

Reported maximum bite forces on anterior teeth are approximately 228 N for men and 198 N for women. The study's outcomes indicate that the fracture resistance of all tested restoration techniques exceeds the typical biting forces applied to anterior teeth. Therefore, while fiber posts provide superior resistance, using reinforced composite cores as combined post-and-core systems for endodontically treated maxillary central incisors may offer sufficient strength for clinical service.

## Data Availability

Available on reasonable request.
